# Hyaluronic Acid Fillers Enriched with Glycine and Proline in Eyebrow Augmentation Procedure

**DOI:** 10.1007/s00266-021-02412-2

**Published:** 2021-07-06

**Authors:** Antonio Scarano, Biagio Rapone, Domenico Amuso, Francesco Inchingolo, Felice Lorusso

**Affiliations:** 1 Department of Medical, Oral and Biotechnological Sciences and CAST, University of Chieti-Pescara, Via Dei Vestini 31, 66100 Chieti, Italy; 2 Department of Basic Medical Sciences, Neurosciences and Sense Organs, “Aldo Moro” University of Bari, 70121 Bari, Italy; 3 Department of Medical, Oral and Biotechnological Sciences, University of Chieti-Pescara, Chieti, Italy; 4 Department of Interdisciplinary Medicine, University of Bari “Aldo Moro”, 70121 Bari, Italy; 5 Department of Medical, Oral and Biotechnological Sciences, University of Chieti-Pescara, Chieti, Italy

**Keywords:** Hyaluronic acid, Dermal filler, Eyebrow, Eyebrow augmentation, Skin rejuvenation

## Abstract

**Background:**

The eyebrow area is a clinically critical district due to the anatomical complexity and the propensity to aging-related atrophy. Hyaluronic acid fillers have been proposed to recover the dermal volume of the facial and lips regions.

**Aim:**

The aim of the present investigation was to evaluate hyaluronic acid fillers enriched with glycine and proline for the treatment of eyebrow augmentation.

**Methods:**

A total of 15 healthy patients were treated with eyebrow augmentation procedure. The distance between mid-bipupil to lateral eyebrow and mid-eyebrow to the medial eyebrow was measured before, immediately after treatment and at follow-up of 6 months.

**Results:**

The healing period was uneventful, and no evidence of inflammation or swelling associated with the treatment was reported. No macroscopical alteration was reported in the surrounding tissues with no evidences of visible wheals or lumps in the treated sites at the follow-up. Before treatment, the angle was equal to 9.32 ± 0.2°, while after treatment it was 11.21 ± 0.4° (*p* < 0.01); after three and 6 weeks, it was, respectively, 10.66 ± 0.2° (*p*<0.05) and 10.02 ± 0.3°(*p* > 0.05).

**Conclusions:**

The study results suggest that the hyaluronic acid fillers enriched with glycine and proline treatment resulted as being a useful procedure for augmentation, contour and volume definition and elevation of the eyebrow region with a high-level aesthetic result.

**Level of Evidence IV:**

This journal requires that authors assign a level of evidence to each article. For a full description of these Evidence-Based Medicine ratings, please refer to the Table of Contents or the online Instructions to Authors www.springer.com/00266.

## Introduction

Facial aging is the result of changes in the soft tissues and bone in the three-dimensional (3-D) topography of the underlying structures and superficial textural wrinkling of the skin [1]. The aging signs of lower, middle and upper third facial are visible in the nose, periauricular, perioral regions and periorbital [2]. Facial changes are caused by atrophy and cutaneous ptosis and by the reduction of the craniofacial bones, and the muscles of facial expression increase the formation of wrinkles through boundaries of the deep mid-facial fat compartments. The eyelid–eyebrow unit plays an important role in facial aesthetics and influences the patient’s self-confidence and the psychological and social quality of life.

The eyes, due to drooping of the forehead and/or brows, can appear more tired due to aging of the tissues which deflate, and the underlying bony orbital rim is exposed [3, 4]. The eyebrow position droops by a few millimetres, especially laterally, and the eyebrow fat pad shrinks, leading to a flatter appearance and loss of convexity and three-dimensional projection [5]. After forty, the appearance ages due to an ever-increasing drooping eyebrow and several studies have described different aesthetic criteria for the ideal eyebrow position and shape. In 1974, Westmore described the ideal eyebrow as represented by a lateral arch with the apex peaking above the lateral limbus of the iris, while the medial and lateral ends of the eyebrow must be at the same height [6]. The perioral and eye are very important for the appearance of patients. The aging of this area is characterized by reduction of the volume and shape, curvature and ptosis which can impair normal peripheral vision [7]. The eyes together with the mouth have important functions related to facial expressions. Interesting study findings suggest that the mouth and eyes are equally important in facial emotion and expressions. For this reason, some patients require rejuvenating of the eye and lip areas. When a facial area is treated with soft tissue augmentation by filler, it appears rejuvenated and usually hyaluronic acid filler is used in the lips to improve their volume and shape, but in the eyes the filler is less used. Different techniques have been used for rejuvenating eyebrows such as botulinum toxin which has been employed for the reduction of periocular rhytides [8, 9]. Hyaluronic acid gel injections have also been used for eyebrow volumizing, to adjust the height, topography and contour of the eyebrows with excellent clinical results [10].

Hyaluronic acid was described by Meyer and Palmer [11] who had isolated it from bovine vitreous humour, the glycosaminoglycan, and it is composed of hyaluronic acid bounded to an amino-sugar [12–15]. It is a biological or synthetic product characterized by a gelatinous or compact consistence that should be applied by injection to the treatment site [16].

Hyaluronic acid is naturally present in the connective tissue of living organisms of different species. It is present in the vitreous humour, synovial fluid and, above all, in the extracellular matrix [17].

Hyaluronic acid differs from most glycosaminoglycans (GAGs) because it does not contain sulphate sugars, the disaccharidic units are identical, and the long length chain not generally covalently joined to any central protein. Moreover, while other GAGs are synthesized inside the cell and released by exocytosis, hyaluronic acid is directly “spun” from the cell surface by an enzymatic complex immersed in the plasma membrane [18]. Cross-linked hyaluronic acid has been proposed for many facial area applications, including eyebrows, for shaping and volumization [19].

The aim of this investigation was to evaluate eyebrow augmentation treated by cross-linked hyaluronic acid.

## Materials and Methods

### Enrolment Patients

The present clinical study was based in a private practice in Montesilvano (Italy), in full accordance with ethical principles, including the World Medical Association Declaration of Helsinki and the additional requirements of Italian law. Furthermore, the University of Chieti-Pescara, Italy, classified the study to be exempt from ethical review as it carries only negligible risk and involves the use of existing data that contains only non-identifiable data about human beings. Informed consent was obtained to publish the information and images in an online open-access publication.

A total of fifteen female healthy patients were treated. All participants were female with mean age 49 ± 3 (age range 47–58 years old) affected by eyebrow hypotonia and reduced volume who were admitted for aesthetic treatment and experimental research. The deflated eyebrows with underlying and exposed bony orbital rim were deemed suitable for study. All subjects signed an informed consent for the research and were treated with eyebrow augmentation procedure.

### Eyebrow Augmentation Procedure

**Fig. 1:**
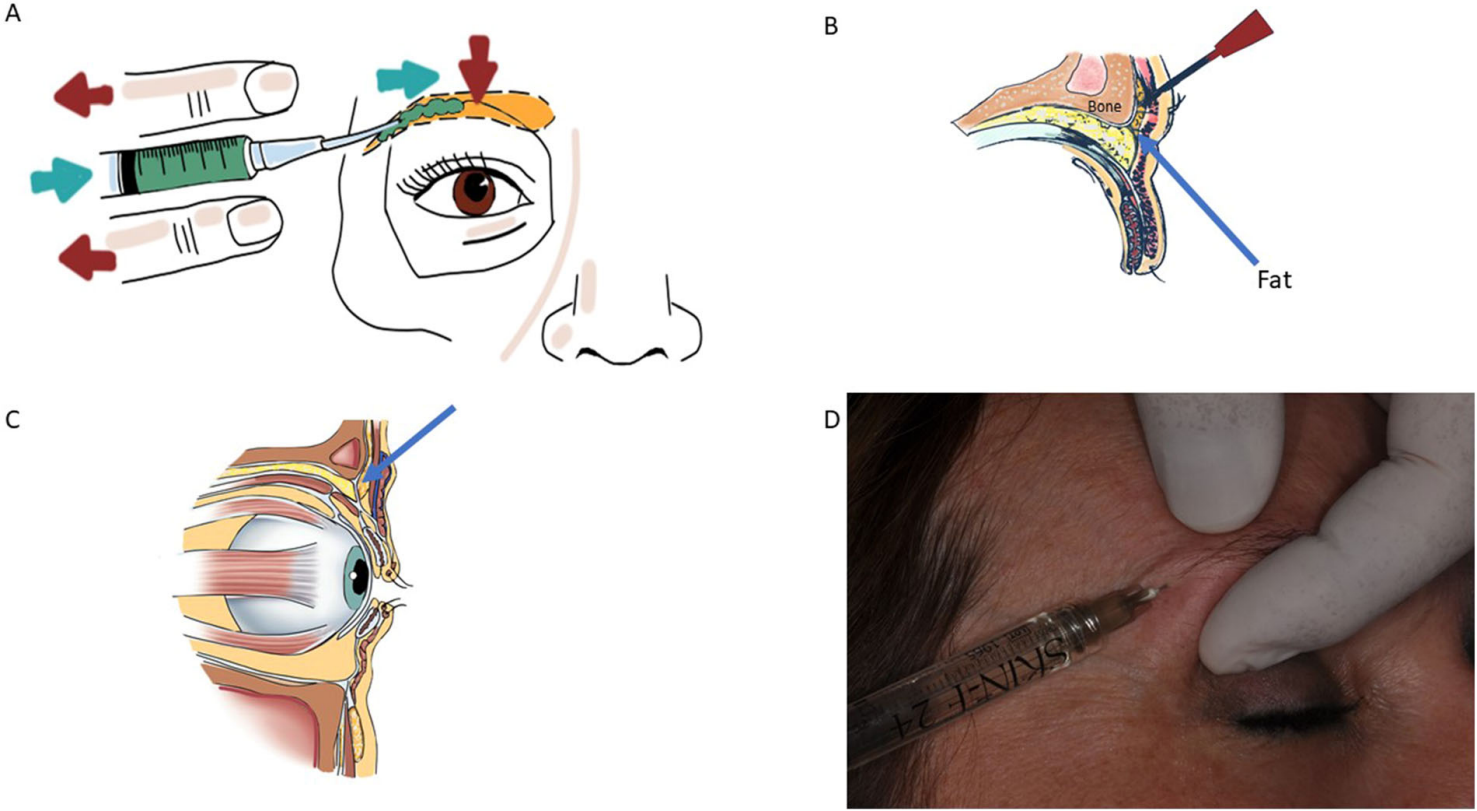
**a** The filler is delivered to the lateral one-third of the eyebrow along the superior orbital rim using a retrograde linear threading technique. **b** The HA is delivered in a preperiosteal plane under retro-orbicularis oculi fat (arrow). **c** Preperiosteal plane under retro-orbicularis oculi fat; the filler is delivered in this area (arrow). **d** Filler delivery to the lateral one-third of the eyebrow along the superior orbital rim using a retrograde linear threading technique

**Fig. 2:**
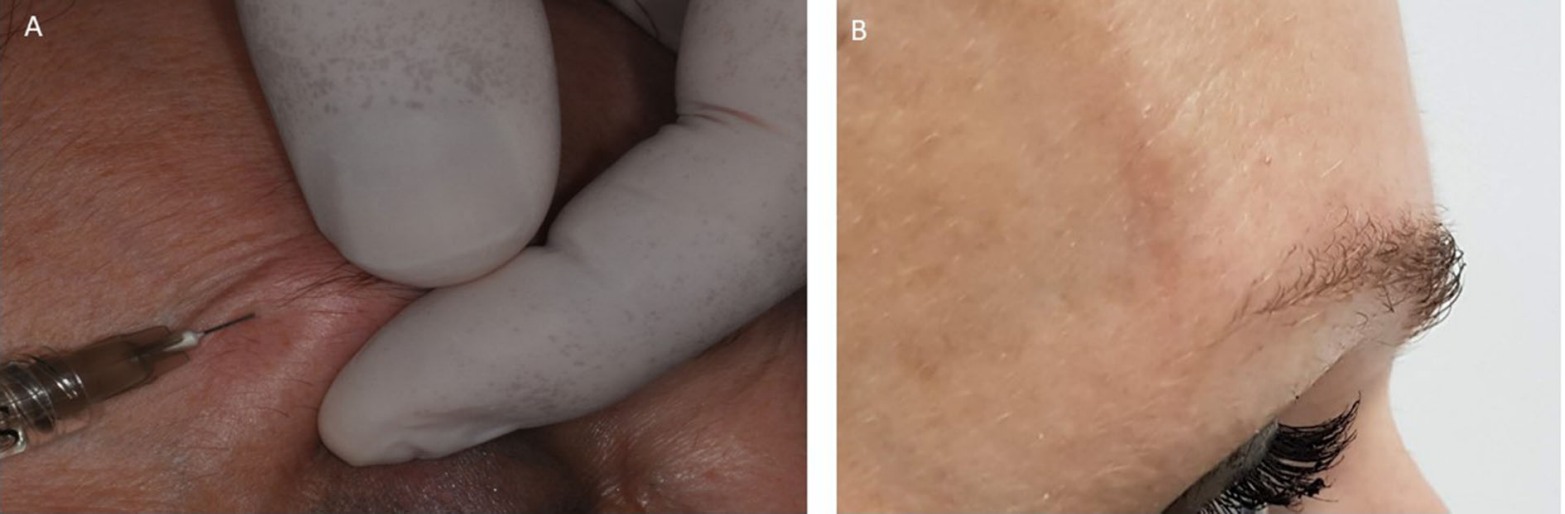
**a** Filler injected in the lateral eyebrow. A small aliquot (0.2–0.3 ml) of filler was placed for each eyebrow until its clinical effect was visible. **a** Immediately after hyaluronic acid injection with anterior projection of the eyebrow

**Fig. 3:**
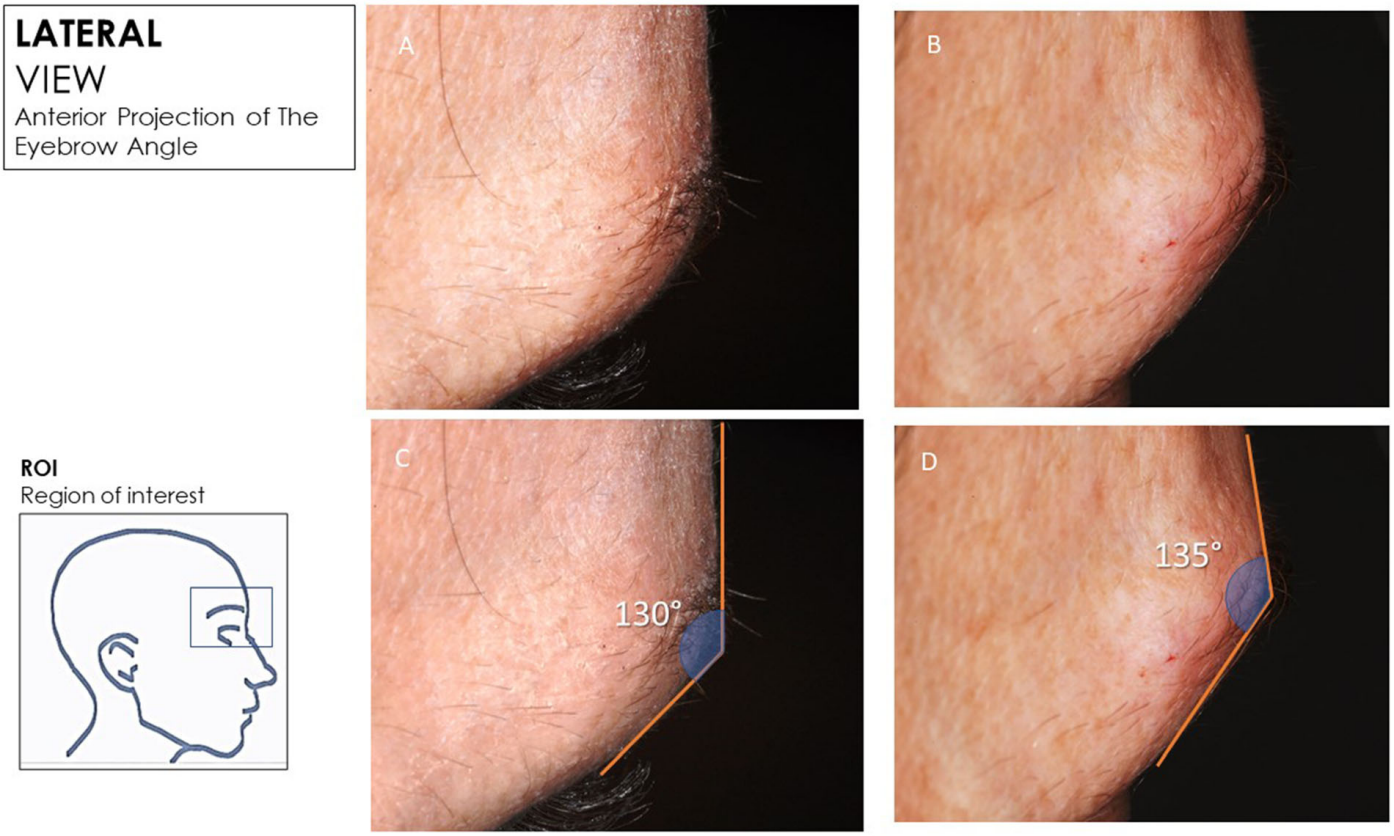
**a**, **b **Before treatment. **c**, **d** Immediately after hyaluronic acid injection with anterior projection of the eyebrow

**Fig. 4:**
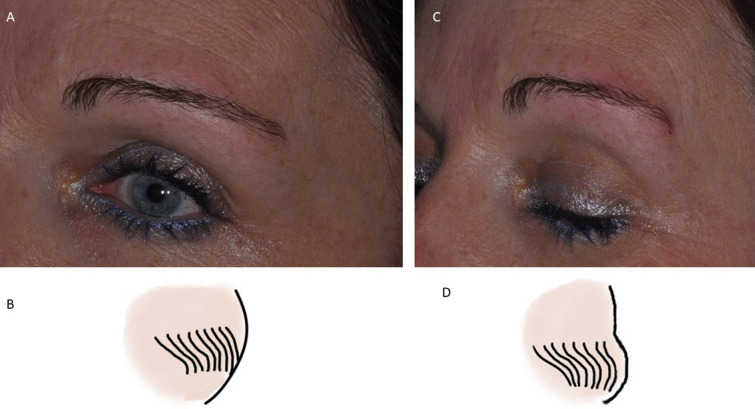
**a**, **b** Before treatment. **c, d** Immediately after hyaluronic acid injection with anterior projection of the eyebrow and improvement of the convexity of eyebrow. **c** Before treatment. **b.** Immediately after treatment with improvement of the elevation of the eyebrow tail (blue arrow). Anterior projection of the eyebrow and improvement of the convexity of the eyebrow

**Fig. 5:**
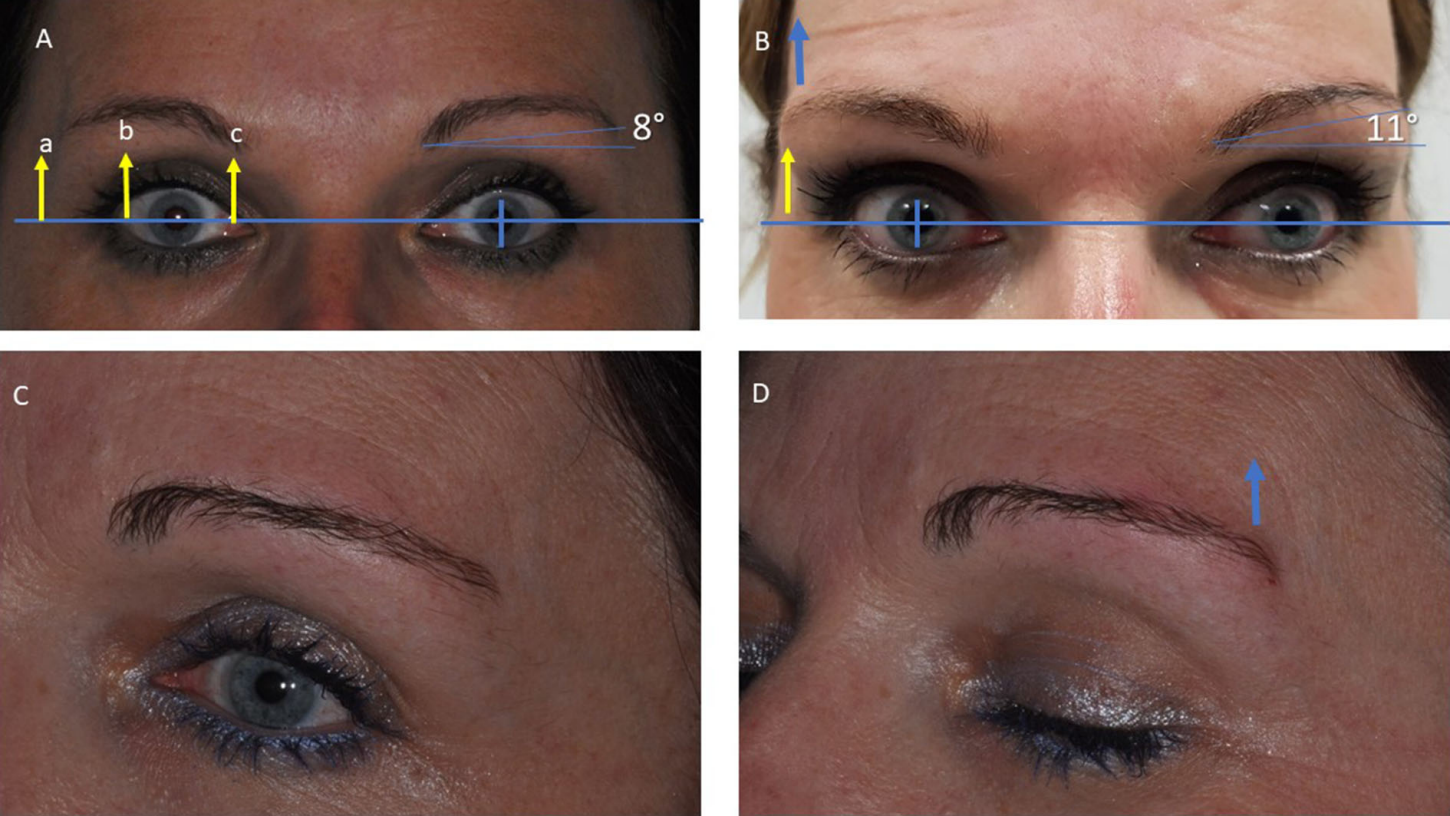
**A** Before treatment. The distances by the line from mid-bipupil (blue line) to lateral eyebrow (point **a**), mid-eyebrow (**b**) and the medial eyebrow (**c**) were evaluated. **B** Immediately after treatment, lateral eyebrow restores the youthful appearance by improving the elevation of the eyebrow tail (blue arrow). **C** Before treatment. **B** Immediately after treatment with improvement of the elevation of the eyebrow tail (blue arrow)

The hyaluronic acid, in the form of cross-linked sodium hyaluronate and 2.5% of amino acids, glycine and proline, in sterile buffered water (Italfarmacia, Rome, Italy), was used in this study. The eyebrow skin was covered with anaesthetic cream containing lidocaine and prilocaine (Emla, AstraZeneca, Svezia) by blocking nerve signals in the treated area. It was applied 30–60 min before the procedure and then removed with gauze, and the skin was disinfected with chlorhexidine 0.2% immediately before the injections. All the treatment was performed by a single aesthetic surgeon. The filler was injected by the use of a 27G needle, length 13 mm, to provide an eyebrow augmentation, and the application was administered following a retrograde linear and bolus application. The HA was delivered to the preperiosteal plane under retro-orbicularis oculi fat (ROOF) to the lateral one-third of the eyebrow along the superior orbital rim using a retrograde linear threading technique (Fig. 1). A small aliquot (0.2–0.3 ml) of filler was placed for each eyebrow until a clinical effect was visible (Fig. 2). Lateral canthus was the point of needle insertion, pushed in for 10–13 mm along the eyebrow; at this point, the HA was delivered to the preperiosteal plane as the needle was removed, determining soft tissue augmentation with retrograde linear threading technique. HA was delivered using only one pass of the needle in linear retrograde technique. Once the needle was in depth between the preperiosteal plane under the sub-orbicularis ROOF plane, the filler was delivered creating a canal-shaped pattern and the delivery of the filler was interrupted immediately before completely withdrawing the needle. No massage was performed, we recommended intermittent local application of ice for 10 min, and no patient needed oral cortisone or anti-inflammatories. Patients were additionally photographed in frontal and oblique views at 1 m (Figs. 2, 3, 4, 5), with standardized zoom and automatic focus, allowing for a clear image of the eyebrows. We evaluated the eyebrow arch by a right-angle triangle with the two catheti that represent the bipupillar and eyebrow line.

The distances of the lines from mid-bipupil to lateral eyebrow, mid-eyebrow and the medial eyebrow were calculated before and after treatment with follow-up of 6 months. The measurements described above were generally performed on both eyes. Also, the angle formed from the mid-bipupil to the lateral eyebrow was calculated before and after treatment (Fig 5). The measurements of the photographs were done by a second blind operator.

### Statistical Analysis

The study data were collected and evaluated by the Graphpad 6 (Prism, San Diego- CA USA) statistical software package. The Shapiro–Wilk test was performed to evaluate the normal distribution of the study data which were normally distributed, and the paired t Student’s test was used to calculate the statistical significance of the average eyebrow position and angle position.

The repeated measures ANOVA followed by Tukey post hoc test was performed to evaluate the average eyebrow height and average eyebrow angle at the different experimental times.

## Results

### Macroscopic Findings

A mild swelling and prominence of the implant were observed as immediate local tissue response. On the following day, no bruising was recorded, and to reduce this incidence, we avoided needle repositioning for more than one passage. Macroscopically, a clinical effect with an appreciable change in eyebrow contour was reported immediately after the procedure (Fig. 2).

Hyaluronic acid deposits were visible at the 90-day follow-up, after which there was a gradual reduction of volume and the aesthetical effect was appreciable for up to 180 days. This aesthetical effect was observed for all patients. The healing period was uneventful, and no local flogistic or oedema evidence was found in the treated region. No macroscopical abnormality was observed in the surrounding tissues, and no cases of visible wheals or lumps were observed. The angle from the mid-bipupil to the lateral brow and tail mid-brow all showed statistically significant decreases compared to the baseline (*p* < 0.05) (Figs. 3, 4). The positions of the lateral eyebrow margin were augmented after filler treatment. The filler of the ptotic lateral eyebrow restores a youthful appearance with anterior projection of the eyebrow and improves the convexity of the eyebrow. The eyebrow was more evenly arched, and the upper eyelid platform was instantaneously enlarged (Fig. 5).

**Fig. 6:**
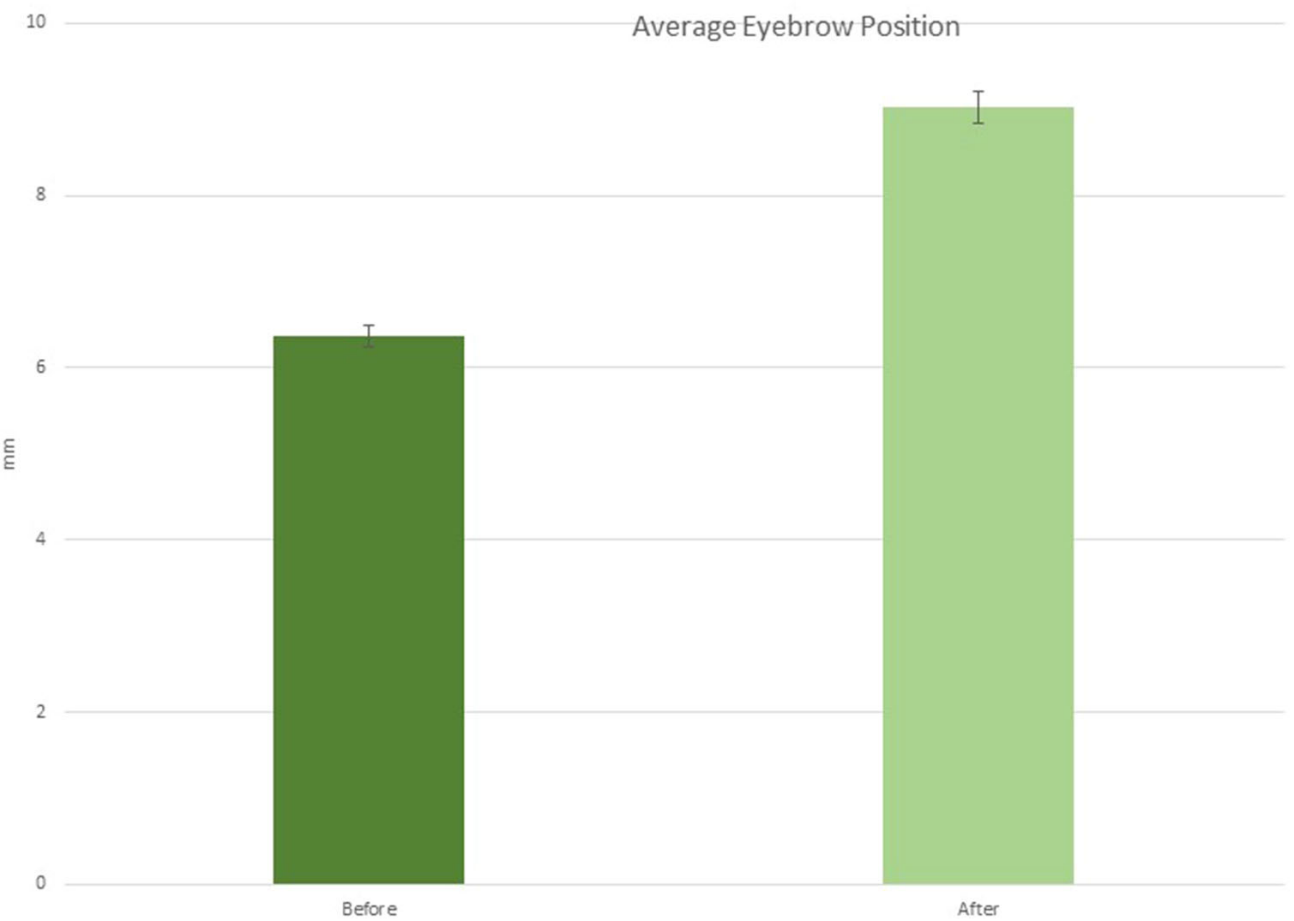
Graph of average eyebrow position before and after the treatment

**Fig. 7:**
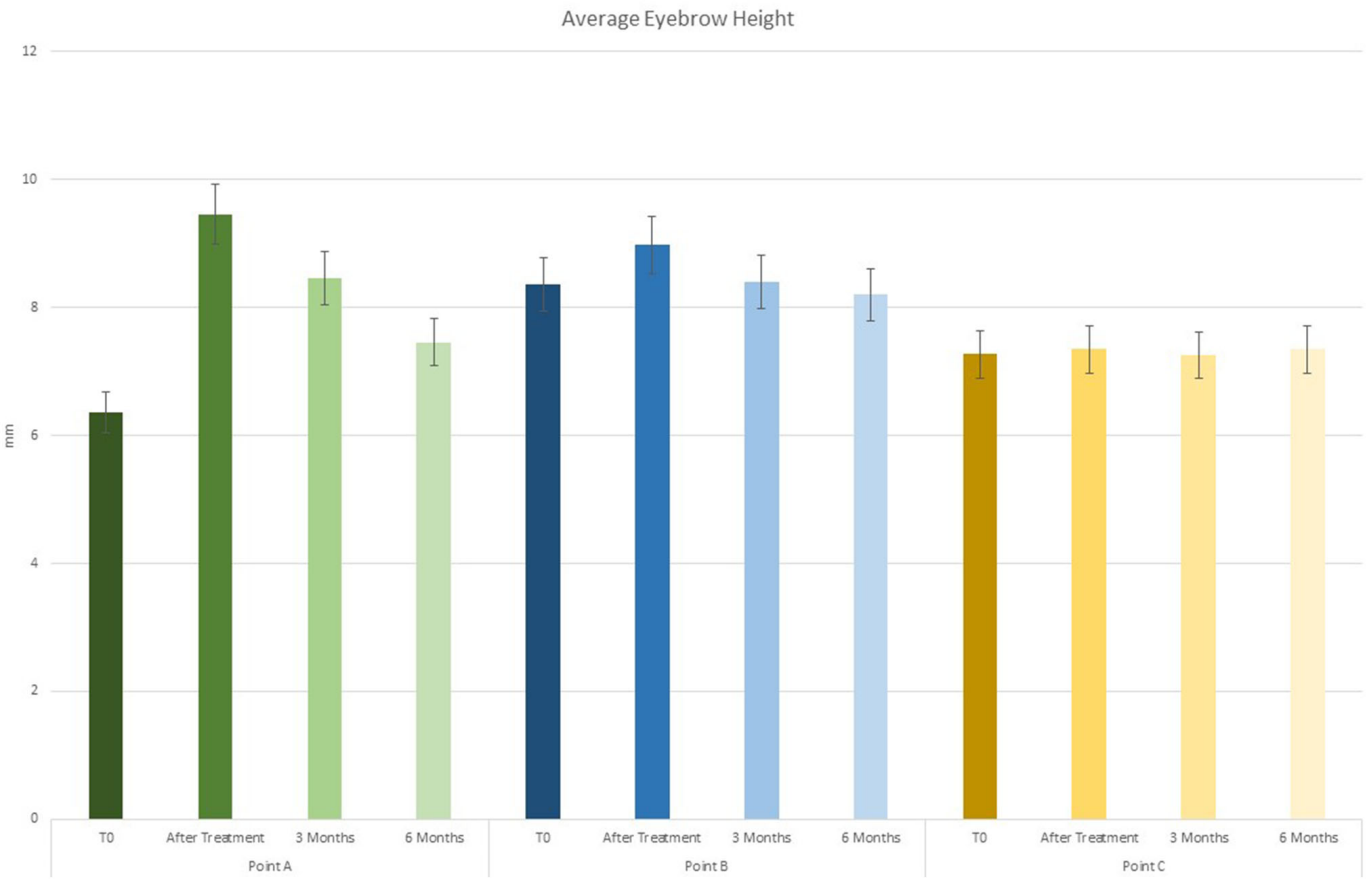
Graph of average eyebrow height at T0, after treatment, at 3 and 6 months for points A, B and C

**Fig. 8:**
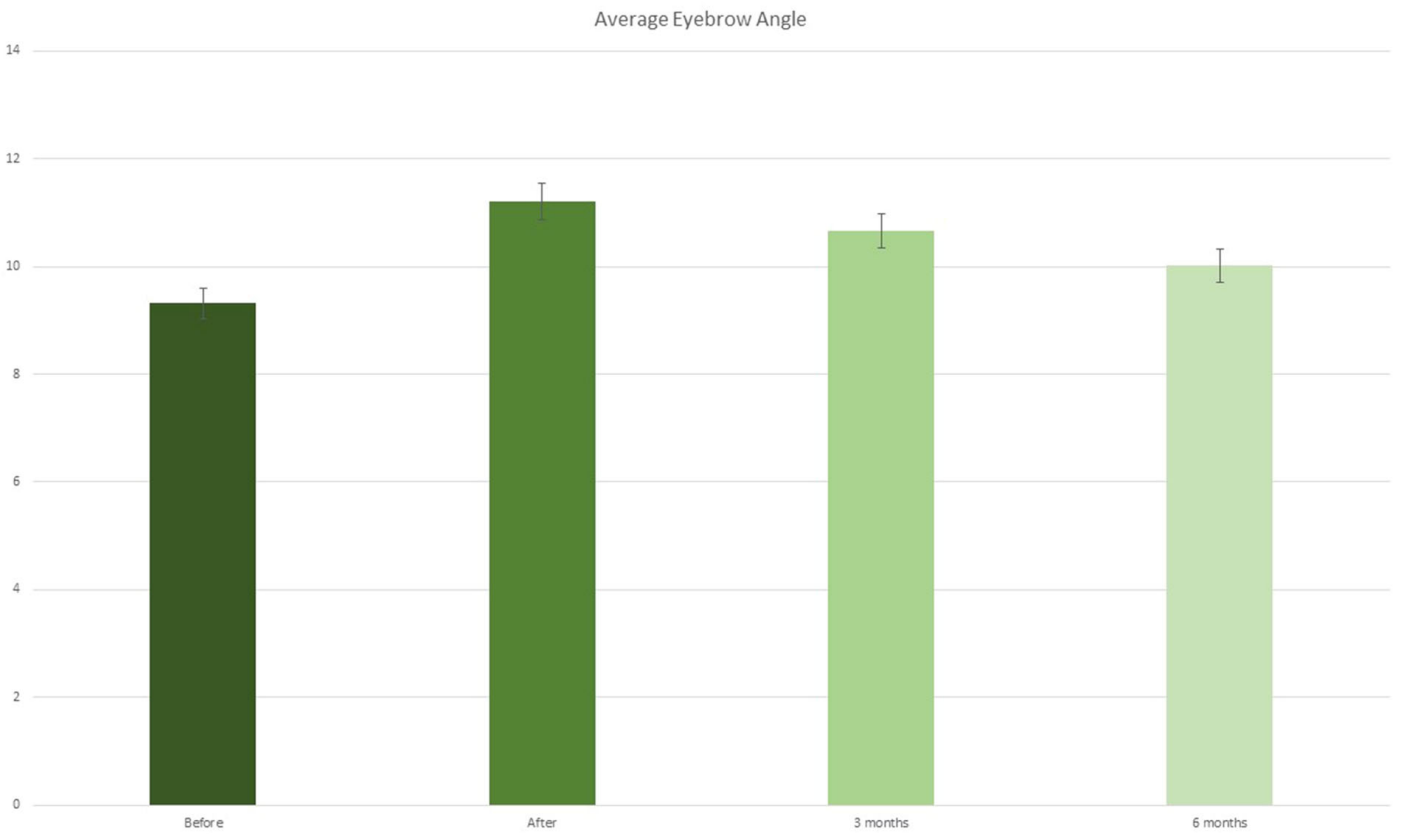
Summary of average eyebrow angle at T0, after treatment, at 3 and 6 months

The average eyebrow height was 6.36 ± 0.2 mm in point A, 8.36 ± 0.2 mm in point B and 7.26 ± 0.3 mm in point C before treatment, while after filler treatment it was 9.46 ± 0.2 mm in point A, 8.98 ± 0.3 mm in point B and 7.35 ± 0.2 mm in point C (Figs. 6, 7, 8).

After 3 months, we recorded eyebrow height 8.46 ± 0.2 mm in point A, 8.40 ± 0.3 mm in point B and 7.25 ± 0.2 mm in point C (Figs. 5, 6, 7, 8). After 6 months, we recorded eyebrow height 7.46 ± 0.2 mm in point A, 8.20 ± 0.2 mm in point B and 7.35 ± 0.2 mm in point C (Table 1).

**Table 1.** Summary of average eyebrow height at T0, after treatment, at 3 and 6 months for points A, B and C

Average eyebrow height	Point A	*P* value	Point B	*P* value	Point C	*P* value
T0	06.36±0.2	*P* < 0.01	08.36±0.2	*P* < 0.05	07.26±0.3	*P* > 0.05
After treatment	09.46±0.2		08.98±0.3		07.35±0.2	
3 months	08.46±0.2	*P* < 0.01	08.40±0.3	*P* < 0.05	07.25±0.2	*P* > 0.05
6 months	07.46±0.2		08.20±0.2		07.35±0.2	

Average eyebrow height	Point A	Point B	Point C
T0	06.36 ± 0.2	08.36 ± 0.2	07.26 ± 0.3
After treatment	09.46 ± 0.2	08.98 ± 0.3	07.35 ± 0.2
3 months	08.46 ± 0.2	08.40 ± 0.3	07.25 ± 0.2
6 months	07.46 ± 0.2	08.20 ± 0.2	07.35 ± 0.2

Before treatment, the angle was equal to 9.32 ± 0.2°, while after treatment it was 11.21 ± 0.4° (*p* < 0.01); after 3 and 6 weeks, it was, respectively, 10.66 ± 0.2° (*p* < 0.05) and 10.02 ± 0.3°(*p* > 0.05) (Figs. 6, 7, 8 and Table 2).

**Table 2.** Summary of average eyebrow angle at T0, after treatment, at 3 and 6 months

Average eyebrow angle	Before	After	3 months	6 months
Mean angle	9.32±0.2°	11.21±0.4°	10.66±0.2°	10.02±0.3°
*P* value	–	*P* < 0.01	*P* < 0.05	*P* > 0.05

### Statistical Analysis

The mean positions of the upper eyebrow and angle before and after filler treatment were increased with statistical difference in point A (*p* < 0.01) with a less significance in point B (*p* < 0.05).

No statistical difference was detected on the position of point C. The mean angle of the upper eyebrow before and after filler treatment was increased with statistical difference. No difference was detected at 6 months after treatment (*p* > 0.05) (Table 2).

## Discussion

The clinical outcomes of the present study showed an augmentation of the eyebrows after use of the hyaluronic acid filler with a rejuvenation of eyebrow contour that shows a fuller eyebrow with lifting of the lateral tail, resulting in a more youthful appearance.

The connective scaffold virtual spaces appeared maintained as well as the surface texture. Where the material was injected, it generated areas with a homogeneously finely fibrillar appearance that was in very close contact with the collagen and elastic fibres. The histological effectiveness of the investigation highlighted the aesthetical and biostimulatory effect produced by injections of hyaluronic acid into tissues, in order to stimulate these tissues to complete the initial action performed by the filler. These intracellular structures are characterized by the emission of lamellar cytoplasmic prolongations and show aspects of nucleocytoplasmic activation constituted by a hypertrophy of the endoplasmic reticulum and of the Golgi complex [20, 21]. The histological results in a previous study showed that this treatment is associated with an increase in collagen and hyaluronic acid that suggests a restoration of the cellular metabolism of the epidermis [22]

Eyebrows play an important role in facial aesthetic perception [23], and their augmentation with HA filler is one technique for improving the facial appearance. Loss of various fat pad volumes and collagen in the dermis can create the appearance of eyebrow drooping.

During aging, there is a reduction of elastin production and collagen, the substance that enables skin to snap back into place. The ratio of type I to type III collagen also decreases in biochemical terms, and the shape of the elastic fibres changes, spreading in a laminar shape between the collagen bundles which appear tiny and fragmented [24]. The filler used in the present research was added with glycine and proline, two amino acid constituents of all the types of collagen, because their endogenous synthesis is inadequate for maximal growth and collagen production [25]. Proline is also a major substrate for the synthesis of arginine for the production of nitric oxide to maintain normal haemodynamics and nutrient transport in the body [26, 27].

A recent photographic study of eyebrow position and shape changes with aging revealed elevation of the medial and central eyebrow compared with the lateral eyebrow [28] for chronic activation of the frontalis muscle [29].

The periocular region is a complex and dynamic part of the face, and different resurfacing techniques are commonly used to alleviate these symptoms by surgical and non-surgical methods. The ptosis of eyebrow treatment was initially primarily surgical, but a surgical lift only elevates the tail of the eyebrow and does not restore its anterior projection. Non-surgical methods are injection of botulinum toxin A, especially for the elevation of the eyebrow tail and glabellar and crow’s feet areas [30]. Atmospheric plasma is a new technique used for rejuvenation of the perioral [31] and periocular areas [32, 33]. Many studies have described the effectiveness of HA in soft tissue augmentation procedures [34–38], but there are less reports in the literature of eyebrow augmentation with HA [39, 40]. In the present study, we observed that the infiltration of HA in the eyebrow determines the volumetric build up and changes the curvature of the eyebrow, bringing it from concave to convex. The use of HA for eyebrow rejuvenation is safe; in fact a study on cadavers showed that highly dense ROOF septal barriers appeared to significantly influence the anatomical position of hyaluronic acid gel [41]. This septal allows to position the HA solely within a preperiosteal plane avoiding migration by dense intra–retro-orbicularis oculi fat (ROOF) septal connective tissue bands.

The clinical and histological outcome of this study confirms the benefit of an HA product injection for eyebrow revenant and augmentation. The HA used for eyebrow augmentation in this study was safe and well tolerated. No severe side effects, such as abnormal eyebrow texture, were observed, preserving the natural movements, firmness and symmetry, function and sensation of the eyebrow, and also there was no mass formation in the treated sites. The clinical results of the present study for eyebrow augmentation with hyaluronic acid filler provided a beneficial, durable treatment and good safety profile. In older patients, the eyebrow tends to lose its lateral arch, and it appears somewhat flattened. The results indicate the methods used for a rejuvenation of eyebrow contour show a fuller eyebrow with lifting of the lateral tail, resulting in a more youthful appearance. The limit of this study was that the aesthetic results achieved with HA enriched with glycine and proline were not compared with HA only. Another limit of this study was the small number of patients treated. So, a comparative future study is important to confirm the outcomes achieved in the present study.

## Conclusion

In conclusion, the study showed that hyaluronic acid mixed with amino acid fillers is a suitable technique for eyebrow augmentation, enhancing eyebrow contour and volume, and may be used for improving the elevation of the eyebrow tail.
